# Distinct Agonist Regulation of Muscarinic Acetylcholine M_2_-M_3_ Heteromers and Their Corresponding Homomers*

**DOI:** 10.1074/jbc.M115.649079

**Published:** 2015-04-27

**Authors:** Despoina Aslanoglou, Elisa Alvarez-Curto, Sara Marsango, Graeme Milligan

**Affiliations:** From the Molecular Pharmacology Group, Institute of Molecular, Cell, and Systems Biology, College of Medical, Veterinary and Life Sciences, University of Glasgow, Glasgow G12 8QQ, Scotland, United Kingdom

## Abstract

Each subtype of the muscarinic receptor family of G protein-coupled receptors is activated by similar concentrations of the neurotransmitter acetylcholine or closely related synthetic analogs such as carbachol. However, pharmacological selectivity can be generated by the introduction of a pair of mutations to produce Receptor Activated Solely by Synthetic Ligand (RASSL) forms of muscarinic receptors. These display loss of potency for acetylcholine/carbachol alongside a concurrent gain in potency for the ligand clozapine N-oxide. Co-expression of a form of wild type human M_2_ and a RASSL variant of the human M_3_ receptor resulted in concurrent detection of each of M_2_-M_2_ and M_3_-M_3_ homomers alongside M_2_-M_3_ heteromers at the surface of stably transfected Flp-In^TM^ T-REx^TM^ 293 cells. In this setting occupancy of the receptors with a muscarinic antagonist was without detectable effect on any of the muscarinic oligomers. However, selective agonist occupancy of the M_2_ receptor resulted in enhanced M_2_-M_2_ homomer interactions but decreased M_2_-M_3_ heteromer interactions. By contrast, selective activation of the M_3_ RASSL receptor did not significantly alter either M_3_-M_3_ homomer or M_2_-M_3_ heteromer interactions. Selectively targeting closely related receptor oligomers may provide novel therapeutic opportunities.

## Teaser

**Background:** Muscarinic receptors can form both homo- and hetero-oligomers.

**Results:** Co-expression of M_2_ and M_3_ receptors resulted in concurrent detection of both homomer and heteromer interactions and regulation of M_2_-containing forms by agonist.

**Conclusion:** Co-existing receptor oligomers display differential regulation.

**Significance:** Oligomers of closely related receptors display distinct properties that may be targeted therapeutically.

## Introduction

Members of the family of muscarinic acetylcholine receptors constitute models for understanding more broadly the superfamily of rhodopsin-like G protein-coupled receptors (GPCRs)^3^ in terms of signaling, structure and pharmacology (1–3). The existence of complexes between muscarinic receptors, in the form of homomers and heteromers has been reported previously (4–9) and the basis and importance of dimerization/oligomerization involving members of this group of GPCRs has been discussed extensively (10–12).

The growing availability of crystal structures of different rhodopsin-like GPCRs has, in many cases, shown potential interaction interfaces between monomeric units (13–15). However, it remains uncertain if these are of physiological significance or simply reflect the most effective way of producing a crystal lattice. Moreover, it is clear that purified and reconstituted monomeric units of such receptors are able to interact with heterotrimeric G proteins in a manner that is regulated by guanine nucleotides and, therefore, in a functionally relevant manner (16–17). In addition to this, there are widely conflicting views on the stability of GPCR-GPCR interactions (18–21), whether this varies substantially within closely related groups of GPCRs, and on the effects or otherwise of receptor ligands on such interactions (see Ref. 11 for review). Furthermore, although it is widely accepted that co-expression of pairs of GPCRs that are able to interact may result in the concurrent presence of each of heteromers containing both GPCRs as well as the corresponding homomers, this has been challenging to demonstrate directly (22). Herein, we use co-expression of forms of the human muscarinic M_2_ and M_3_ receptors to explore these issues. We demonstrate concurrent detection of M_2_-M_2_, M_3_-M_3_, and M_2_-M_3_ interactions at the surface of cells and distinct agonist regulation of these interactions.

## Experimental Procedures

### 

#### 

##### Materials

Materials for cell culture were from Sigma Aldrich or Life Technologies unless otherwise stated. Clozapine N-oxide (CNO) was from Enzo Life Sciences. Carbachol and atropine were from Sigma-Aldrich. Immunological reagents able to identify the epitope tags were obtained from New England Biolabs (anti-SNAP) or Roche (anti-HA). The antiserum directed against VSV epitope was produced in-house. All secondary IgG, horseradish peroxidase-linked antibodies were from GE Healthcare. The radioligand [^3^H]quinuclidinylbenzilate ([^3^H]QNB) was from PerkinElmer. Flp-In^TM^ T-REx^TM^ 293 cells were from Life Technologies.

##### Molecular Constructs

Generation of the human (h)M_3_RASSL mutant was described by Ref. 4. HA-CLIP-hM_3_RASSL and VSV-SNAP-hM_2_WT cDNA constructs were produced by introducing the metabotropic glutamate 5 receptor (mGluR5) signal sequence followed by either the VSV and SNAP tags or the hemaglutinin (HA) and CLIP tags into the N terminus of the hM_2_WT or hM_3_RASSL receptor, respectively (4, 23).

##### Generation of Flp-In^TM^ T-REx^TM^ 293 Cells Stably Expressing Muscarinic Receptor Constructs

Cells were maintained in complete Dulbecco's modification of Eagle's medium (DMEM) without sodium pyruvate, 4500 mg·l^−1^ glucose, and l-glutamine, supplemented with 10% (*v*/*v*) fetal bovine serum, 1% (*v*/*v*) penicillin/streptomycin mixture, 200 μg·ml^−1^ hygromycin B, and 10 μg·ml^−1^ blasticidin in a humidified atmosphere. Single stable Flp-In^TM^ T-REx^TM^ 293 cell lines able to inducibly express the different cDNA constructs were generated as described previously (4, 22–23). To constitutively co-express a second receptor construct in these cells they were transfected with the appropriate cDNA construct, as described above, and antibiotic-resistant clones selected using 1 mg·ml^−1^ G418. All such cell lines were initially screened by fluorescence microscopy for receptor expression based on covalent binding of SNAP- or CLIP-tagged fluorophores and subsequently by measuring specific binding of [^3^H]QNB in cell membrane preparations.

##### Cell Membrane Preparations

Cells treated or not with doxycycline, were harvested after 24 h, in ice-cold phosphate-buffered saline (PBS) and pellets were frozen at −80 °C for a minimum of 1 h. Pellets were thawed and resuspended in ice-cold 10 mm Tris, 0.1 mm EDTA, pH 7.4 (TE) buffer, supplemented with Complete^TM^ protease inhibitor mixture (Roche Diagnostics). Cells were passed through a 25-gauge needle (5–10 times) and then homogenized on ice, by 50 strokes in a glass-on-teflon homogenizer. Homogenized cells were centrifuged at 200 × *g* for 5 min at 4 °C. The supernatant fraction was removed and transferred to microcentrifuge tubes and subjected to further centrifugation at 90,000 × *g* for 45 min at 4 °C. The pellets were resuspended in TE buffer, and protein concentration was assessed. Membrane preparations were either used directly or kept at −80 °C until required.

##### Radioligand Binding Studies

Binding using various concentrations of [^3^H]QNB was carried out using 5 μg of membrane protein per reaction in assay buffer (20 mm HEPES, 100 mm NaCl, 10 mm MgCl_2_, pH 7.4). Nonspecific binding was defined in the presence of 10 μm atropine. Reactions were incubated for 2 h at 30 °C. Bound ligand was separated from free by vacuum filtration through GF/C filters (Brandel Inc.). The filters were washed twice with assay buffer, and bound ligand was estimated by liquid scintillation spectrometry.

##### Cell Lysate Preparation and Immunoblotting

Cells were harvested, washed twice in ice cold PBS, and pelleted by centrifugation. The pellets were resuspended in radio-immunoprecipitation buffer (50 mm HEPES, 150 mm NaCl, 1% Triton X-100, 0.5% sodium deoxycholate, 10 mm NaF, 5 mm EDTA, 10 mm NaH_2_PO_4_, 5% ethylene glycol, pH 7.4), supplemented with Complete^TM^ protease inhibitors mixture. Resuspended cells were then placed on a rotating wheel for 30 min at 4 °C, and subsequently centrifuged at 21,000 × *g*, for 15 min at 4 °C. Supernatants were collected, and the protein concentration of the lysates determined. Samples were heated at 60–65 °C in 1× Laemmli buffer (10% w/v SDS, 10 mm dithiothreitol, 20% *v*/*v* glycerol, 0.2 m Tris-HCl, 0.05% *w*/*v* bromphenol blue, pH 6.8). The required amount of protein lysate was then loaded on 4–12% NuPAGE^TM^ Novex® Bis-Tris gels (Life Technologies). Following electrophoresis, proteins were transferred onto a nitrocellulose membrane, blocked, and subsequently incubated with the primary antibody/antiserum in 5% fat-free milk TBST (2 mm Tris-base, 15 mm NaCl, and 0.1% *v*/*v* Tween 20, pH 7.4) at 4 °C, overnight. After 5 × 5 min washing steps with TBST, the appropriate horseradish peroxidase-conjugated IgG secondary antibody was incubated with the membrane at room temperature for 1 h. Immunoblots were developed using enhanced chemiluminescence solution (Pierce).

##### Epifluorescence Imaging of Living Cells

Cells were seeded on poly-d-lysine pre-coated cover slips (0.0 mm thickness) to 500,000 cells per cover slip and incubated overnight in the presence or absence of doxycycline in complete DMEM. Cells that expressed HA-CLIP-hM_3_RASSL receptor were labeled with 5 μm CLIP-Surface 488 while those expressing VSV-SNAP-hM_2_WT were labeled using 5 μm SNAP-Surface 549 (New England Biolabs) in complete DMEM for 30 min at 37 °C in 5% CO_2_. Cells were washed three times with complete DMEM and once with HEPES physiological saline solution (130 mm NaCl, 5 mm KCl, 1 mm CaCl_2_, 1 mm MgCl_2_, 20 mm HEPES, pH 7.4, and 10 mm d-glucose). Cover slips were imaged using an inverted Nikon TE2000-E microscope (Nikon Instruments, Melville, NY) equipped with a 40× (numerical aperture-1.3) oil-immersion Pan Fluor lens and a cooled digital Photometrics Cool Snap-HQ charge-coupled device camera (Roper Scientific, Trenton, NJ).

##### Homogeneous Time-resolved FRET (htrFRET)

Cells were grown to 100,000 per well on poly-d-lysine pre-treated 96-well solid black bottom plates (Greiner Bio-One). Cells were induced with doxycycline at the stated concentration for 24 h to express the receptor(s) of interest. After 24 h induction, cell surface receptor expression was monitored by adding 10 nm SNAP-Lumi4Tb or 20 nm CLIP-Lumi4Tb. After incubation at 37 °C/5% CO_2_ for 1 h, cells were washed three times with labeling medium (Cisbio Bioassays), and the fluorescence output was read at 620 nm using a PheraStar FS (BMG Lab technologies).

In htrFRET experiments various combinations of energy donor:acceptor were used to detect either homomers or heteromers. Detection of hM_2_WT homomers was carried out by labeling with 5 nm SNAP-Lumi4Tb with varying concentrations of SNAP-Red. hM_3_RASSL homomers were detected by labeling with 10 nm CLIP-Lumi4Tb and varying concentrations of CLIP-Red. Heteromeric interactions between hM_2_WT and hM_3_RASSL were detected using 5 nm SNAP-Lumi4Tb with varying concentrations of CLIP-Red, or the reverse combination, 10 nm CLIP-Lumi4Tb with varying concentrations of SNAP-Red. Labeling reactions were carried out for 1 h at 37 °C/5% CO_2_. Cells were then washed three times with 100 μl per well labeling medium and plates were either read directly after this or further processed to test the effect of receptor ligands. For the latter experiments, ligands were added to the plates after the washing step and subsequently incubated at the noted temperature and times prior to measurements using a PheraStar FS. Both the emission signal from the SNAP-Lumi4Tb or CLIP-Lumi4Tb (620 nm) and the FRET signal emanating from the acceptor SNAP-Red or CLIP-Red (665 nm) were recorded. Specific 620 nm fluorescence, 665 nm FRET or 665:620 ratio values are shown as the difference between signals obtained from induced and un-induced cells.

##### Triple Labeling htrFRET

Cells were plated, grown, and treated with doxycycline in the same way as described in the previous section. The cells were then simultaneously labeled with three different, but spectrally compatible htrFRET substrates. One donor was used at a time, either SNAP-Lumi4Tb (5 nm) or CLIP-Lumi4Tb (10 nm), in combination with SNAP-Green (100 nm) and CLIP-Red (100 nm). The substrates were prepared at 3× the final concentrations in labeling buffer and 25 μl of each was added per well. Cells were incubated for 1 h at 37 °C/5% CO_2_ and washed three times with labeling buffer. Ligands were added to the plates and incubated at the set time points after which the plates were read using a PheraStar FS. Two different protocols were used to measure the fluorescence output corresponding to energy transfer to the two acceptors, CLIP-Red at 665 nm and SNAP-Green at 520 nm. The donor emission at 620 nm originating from either SNAP-Lumi4Tb or CLIP-Lumi4Tb was also measured in both protocols.

##### Inositol Monophosphate Accumulation Assay

A suspension of 10,000 cells per assay point was prepared in stimulation buffer (10 mm HEPES, 1 mm CaCl_2_, 0.5 mm MgCl_2_, 4.2 mm KCl, 146 mm NaCl, 5.5 mm glucose, and 50 mm LiCl, pH 7.4) and incubated with ligands for 1 h at 37 °C/5% CO_2_ in a white Proxiplate-384 Plus (PerkinElmer). After stimulation, cells were lysed in a mixture of detection reagents prepared in lysis buffer according to the manufacturer's instructions (IP-One Tb kit, Cisbio Bioassays) and incubated for a further hour at room temperature. htrFRET was then measured using a PheraStar FS and changes in inositol monophosphate levels were calculated as ratio of 665/620 nm signals.

##### cAMP Inhibition Assay

A suspension of 4,000 cells per assay point was prepared in Hank's Balanced Salt Solution (HBSS). Cells were co-incubated with forskolin (5 μm) and ligands for 30 min in a white Proxiplate-384 Plus. This step was followed by lysis of cells using a mixture of detection reagents prepared in lysis buffer according to manufacturer's instructions (cAMP dynamic 2 kit, Cisbio Bioassays) and incubation for 1 h at room temperature. htrFRET was measured on a PheraStar FS and the reduction of cAMP levels was calculated as ratio of 665/620 nm.

## Results

**FIGURE 1.**
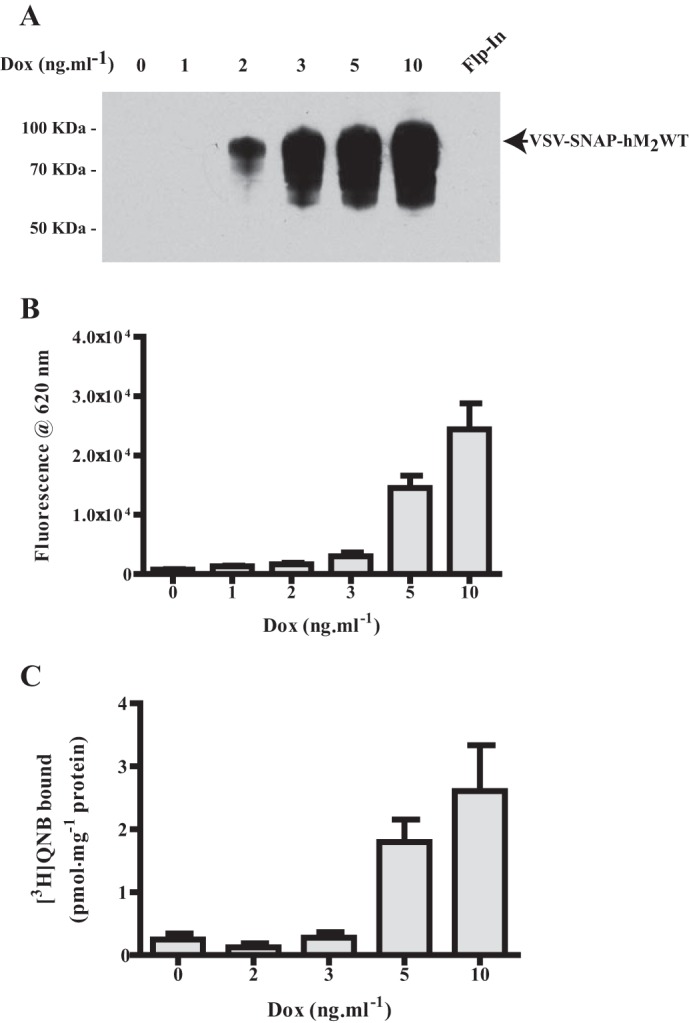
**Inducible control of hM_2_WT receptor expression.** VSV-SNAP-hM_2_WT was cloned into the Flp-In^TM^ TREx^TM^ locus of Flp-In^TM^ TREx^TM^ 293 cells and a population of stably transfected cells isolated. *A*, lysates of these cells, maintained for 24 h in the absence (*0 Dox*) or presence of the indicated concentration of doxycycline, were prepared and resolved by SDS-PAGE. Lysate of parental non-transfected Flp-In^TM^ TREx^TM^ 293 cells (*Flp-In*) provided a negative control. Immunoblotting with an anti-SNAP/CLIP antiserum identified the receptor construct. *B*, intact Flp-In^TM^ TREx^TM^ 293 cells harboring VSV-SNAP-hM_2_WT and treated for 24 h with the indicated concentration of doxycycline were treated with SNAP-Lumi4Tb (5 nm). Following washing, fluorescence emission at 620 nm following excitation at 337 nm defined the relative expression levels of VSV-SNAP-hM_2_WT at the cell surface. *C*, membrane preparations from cells as in *B* were used to define the specific binding of [^3^H]QNB (0.16–0.45 nm in individual experiments). Data are means ± S.E., *n* = 3.

To explore aspects of the potential oligomerization of the wild type (WT) human (h) muscarinic M_2_ acetylcholine receptor, a construct (VSV-SNAP-hM_2_WT) was generated in which the extracellular N-terminal domain was modified to incorporate both the VSV peptide epitope tag and the SNAP protein tag sequences. This was cloned into the doxycycline-inducible locus of Flp-In^TM^ T-REx^TM^ 293 cells and a transfected population selected. Doxycycline-regulated expression of this construct was assessed in three distinct ways. Firstly, immunoblotting with an anti-SNAP/CLIP antiserum of SDS-polyacrylamide gel electrophoresis (SDS-PAGE) resolved lysates of cells that had been maintained for 24 h in the presence of different concentrations of doxycycline identified specific induction of the receptor construct as a polypeptide with apparent molecular mass in the region of 80 kDa (Fig. 1*A*). No equivalent species was detected either in lysates of these cells grown in the absence of doxycycline or in lysates of parental, non-transfected Flp-In^TM^ T-REx^TM^ 293 cells (Fig. 1*A*). Secondly, doxycycline-induced expression and effective cell surface delivery of the construct was defined by fluorescence emission at 620 nm following excitation at 337 nm, subsequent to adding the SNAP-tag label SNAP-Lumi4Tb to intact cells. This reflects covalent attachment of the label to the SNAP tag of the construct. This is located in the extracellular milieu because the N-terminal domain of cell surface targeted GPCRs is anticipated to be outside the cell (Fig. 1*B*). Third, specific binding of concentrations of the muscarinic antagonist [^3^H]QNB, close to the *K_d_* as assessed in saturation binding studies (0.30 ± 0.07 nm, mean ± S.E., *n* = 4), to membranes of doxycycline-induced VSV-SNAP-hM_2_WT cells generated a qualitatively similar profile as labeling of the construct with SNAP-Lumi4Tb (Fig. 1*C*).

**FIGURE 2.**
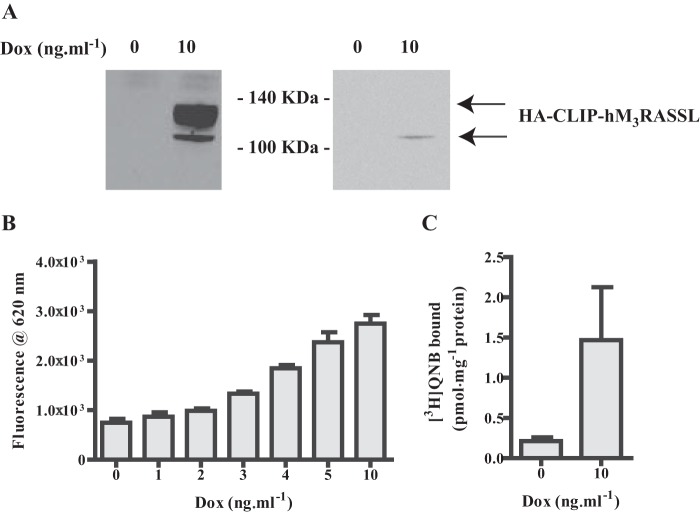
**Characterization of hM_3_RASSL expression.** HA-CLIP-hM_3_RASSL was cloned into the Flp-In^TM^ TREx^TM^ locus of Flp-In^TM^ TREx^TM^ 293 cells and a population of stably transfected cells isolated. *A*, lysates of these cells, maintained for 24 h in the absence or presence of 10 ng·ml^−1^ doxycycline, were prepared and resolved by SDS-PAGE. These were then immunoblotted with either anti-SNAP/CLIP (*left hand side*) or anti-HA (*right hand side*). See “Results” for further details. *B*, akin to Fig. 1 intact Flp-In^TM^ TREx^TM^ 293 cells harboring HA-CLIP-hM_3_RASSL and treated for 24 h with the indicated concentrations of doxycycline were treated with CLIP Lumi4Tb (10 nm). After washing, fluorescence emission at 620 nm following excitation at 337 nm defined the relative expression levels of HA-CLIP-hM_3_RASSL at the cell surface. *C*, membrane preparations from cells as in *B* were used to define the specific binding of [^3^H]QNB (14–20 nm in individual experiments). Data are means ± S.E., *n* = 3. *Note:* the substantially higher concentration of [^3^H]QNB used than in Fig. 1 reflects the loss in binding affinity of antagonists associated with the RASSL version of hM_3_.

**FIGURE 3.**
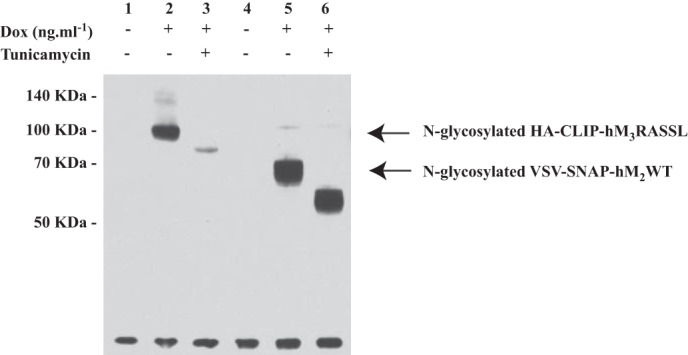
**Characterization of the *N*-glycosylation status of hM_2_ and hM_3_ constructs.** Cells as in Figs. 1 and 2 harboring HA-CLIP-hM_3_RASSL (*1–3*) or VSV-SNAP-hM_2_WT (*4–6*) were uninduced (*1, 4*) or treated with doxycycline to induce the relevant receptor construct (*2–3, 5–6*). Certain cells (*3, 6*) were also grown in the presence of tunicamycin (6 μm) over the entire period of doxycycline induction. Lysates from these cells were resolved by SDS-PAGE and immunoblotted with an anti-SNAP/CLIP antiserum.

We have previously characterized VSV- and SNAP-tagged forms of both the WT muscarinic hM_3_ acetylcholine receptor and a chemically engineered, Receptor Activated Solely by Synthetic Ligand (RASSL) variant (4, 23–24). This form is not able to bind or respond effectively to acetylcholine or related synthetic analogs. Rather, it is activated by the usually inert chemical ligand clozapine N-oxide (CNO) (23–24). Now, a modification of this construct to generate HA-CLIP-hM_3_RASSL in which the N-terminal VSV- and SNAP-tags were replaced with the HA peptide epitope tag and the CLIP protein tag sequence was generated. This was also cloned into the doxycycline-inducible locus of Flp-In^TM^ T-REx^TM^ 293 cells. Doxycycline-induced expression and cell surface delivery of this construct was also characterized by immunoblotting to detect each of the CLIP- and HA-tags (Fig. 2*A*) and, now, by the binding of CLIP-Lumi4Tb (Fig. 2*B*). As anticipated from the substantially larger third intracellular loop of the hM_3_ receptor compared with hM_2_, the apparent molecular mass of the predominant form of HA-CLIP-hM_3_RASSL identified by the SNAP/CLIP antiserum was in the region of 110 kDa (Fig. 2*A*). Such RASSL forms of muscarinic receptors display modestly reduced affinity for many antagonist ligands (24), including [^3^H]QNB, compared with the equivalent WT receptor. Preliminary studies indicated the *K_d_* of [^3^H]QNB for HA-CLIP-hM_3_RASSL to be in the region of 2.5 nm. Therefore, by measuring the specific binding of a substantially higher concentration of [^3^H]QNB (15 nm) than used for VSV-SNAP-hM_2_WT it was also possible to quantify expression of HA-CLIP-hM_3_RASSL (Fig. 2*C*). Noticeably, although the anti-SNAP/CLIP antiserum identified two forms of HA-CLIP-hM_3_RASSL, the HA antiserum identified only the more rapidly migrating and less prominent form (Fig. 2*A*). Pre-treatment of cells during the period of receptor induction with the *de novo N*-glycosylation inhibitor tunicamycin demonstrated the form with lower mobility, which was not identified by the anti-HA antiserum, to be the mature *N*-glycosylated form. Moreover, equivalent studies indicated that VSV-SNAP-hM_2_WT was also *N*-glycosylated in the absence of tunicamycin treatment (Fig. 3) and that these mature forms of the receptors were the predominant species present.

**FIGURE 4.**
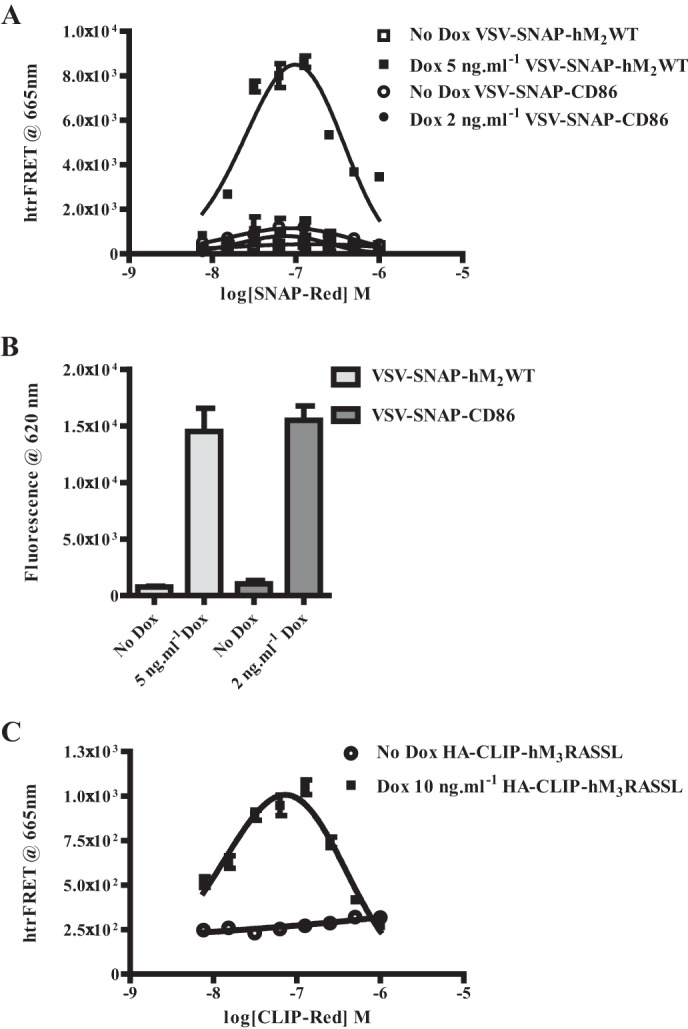
**Detection of hM_2_WT and hM_3_RASSL homo-oligomers in cells induced to express either receptor individually.** *A* and *B*, cells, as in Fig. 1, able to express VSV-SNAP-hM_2_WT in a doxycycline-inducible fashion (squares (*A*), dark bars (*B*)), were compared with equivalent cells able to express VSV-SNAP-CD86 upon addition of doxycycline (circles (*A*), light bars (*B*)). *A*, htrFRET was measured following addition of SNAP-Lumi4Tb (10 nm) and the indicated range of concentrations of SNAP-Red in both uninduced cells (*No Dox, open symbols*) and in cells induced to express the constructs by growth in the presence of the indicated concentrations of doxycycline (*filled symbols*). *B*, these concentrations of doxycycline resulted in cell surface expression of the same level of VSV-SNAP-hM_2_WT and VSV-SNAP-CD86 as detected by fluorescence at 620 nm after excitation at 337 nm that reflects binding of added SNAP-Lumi4Tb (10 nm). *C*, cells able to express HA-CLIP-hM_3_RASSL were uninduced (*No Dox, open symbols*) or treated for 24 h with the indicated concentration of doxycycline to induce the receptor construct (*filled symbols*). These were exposed to a combination of 20 nm CLIP Lumi4Tb and varying concentrations of CLIP-Red. Following excitation at 337 nm, fluorescence emission at 665 nm was assessed as a measure of htrFRET.

In cells induced to express VSV-SNAP-hM_2_WT addition of a single concentration of SNAP-Lumi4Tb, as potential energy donor, along with varying concentrations of SNAP-Red, as potential energy acceptor, generated bell-shaped homogeneous time-resolved (htr)FRET signals. These were detected as emission at 665 nm following excitation at 337 nm and are consistent with VSV-SNAP-hM_2_WT existing, at least in part, as cell surface homo-dimers/oligomers (Fig. 4*A*) (4). By contrast, no such signals were produced in the absence of doxycycline-induced receptor expression (Fig. 4*A*). To define that these htrFRET signals reflected relevant homomeric protein-protein interactions, and not simply proximity due to the level of receptor expression causing crowding or bystander effects, we performed equivalent experiments in Flp-In^TM^ T-REx^TM^ 293 cells able to express the monomeric transmembrane protein CD86 (25) (Fig. 4*A*). This polypeptide was also modified to introduce both the VSV- and SNAP-tag sequences into the extracellular N-terminal domain. Here, addition of a combination of SNAP-Lumi4Tb and varying concentrations of SNAP-Red did not result in significant htrFRET signal in cells induced to express VSV-SNAP-CD86. Indeed, the signal was indistinguishable from cells in which expression of this construct was not induced (Fig. 4*A*). These experiments were carefully designed to result in cell surface expression of the same amount of VSV-SNAP-CD86 as VSV-SNAP-hM_2_WT. This was measured directly by the level of binding of SNAP-Lumi4Tb to each of the receptors, as in Fig. 1*B*, as fluorescence at 620 nm following excitation as 337 nm (Fig. 4*B*). Therefore, VSV-SNAP-hM_2_WT is present within homo-oligomers at expression levels in which such signals are not produced by a well characterized monomeric protein.

Addition of a single concentration of CLIP-Lumi4Tb, as potential energy donor, along with varying concentrations of CLIP-Red to cells induced to HA-CLIP-hM_3_RASSL also generated bell-shaped htrFRET signals (Fig. 4*C*). This was lacking in cells not induced to express HA-CLIP-hM_3_RASSL (Fig. 4*C*). These results also are consistent with homo-dimeric/oligomeric HA-CLIP-hM_3_RASSL interactions (Fig. 4*C*), confirming previous reports of hM_3_-hM_3_ interactions (4).

**FIGURE 5.**
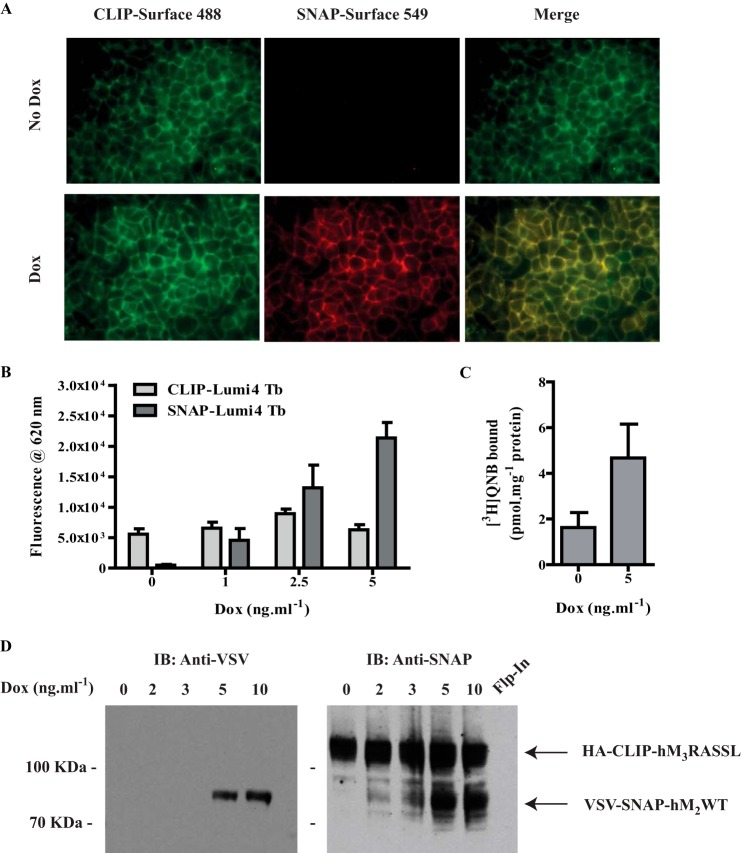
**Characterization of cells able to co-express hM_2_WT and hM_3_RASSL.** Cells, as in Fig. 1, able to express VSV-SNAP-hM_2_WT in a doxycycline-dependent fashion were further transfected with HA-CLIP-hM_3_RASSL and clones expressing this receptor construct isolated. One specific clone is detailed. *A*, cells were maintained in the absence or presence of 5 ng·ml^−1^ doxycycline for 24 h and then either of the cell impermeable dyes, SNAP-surface 549 (to label cell surface hM_2_WT) and CLIP-surface 488 (to label cell surface hM_3_RASSL), was added and the cells imaged. Where indicated the images corresponding to SNAP-surface 549 and CLIP-surface 488 labeling were merged. *B*, cells were maintained in the absence or presence of varying concentrations of doxycycline for 24 h. Subsequently either SNAP Lumi4Tb (*open bars*) or CLIP Lumi4Tb (f*illed bars*) was added and fluorescence emission at 620 nm after excitation at 337 nm was measured to assess relative levels of cell surface VSV-SNAP-hM_2_WT and HA-CLIP-hM_3_RASSL (*n* = 6–8 for each doxycycline concentration). *C*, specific [^3^H]QNB binding to membranes from cells maintained in the absence or presence of 5 ng·ml^−1^ doxycycline was assessed. *Note:* individual experiments were performed with 16–21.6 nm [^3^H]QNB to allow effective detection of hM_3_RASSL as well as hM_2_WT and resulted in poorer data quality due to the relatively poor specific to nonspecific binding ratios at these high concentrations of [^3^H]QNB (means ± S.E., *n* = 4). *D*, immunoblots were performed on membranes prepared from either these cells maintained in the presence of the indicated concentrations of doxycycline for 24 h or from parental Flp-In^TM^ TREx^TM^ 293 cells (*Flp-In*). Panels display anti-VSV (hM_2_WT) (*left hand panel*) or anti-SNAP/CLIP (both hM_2_WT and hM_3_RASSL) (*right hand panel*) immunoreactivity.

To explore the potential for co-expressed hM_2_ and hM_3_ to exist within heteromeric complexes, cells able to express VSV-SNAP-hM_2_WT only following addition of doxycycline, were further transfected with HA-CLIP-hM_3_RASSL and clones constitutively expressing this receptor variant isolated. A substantial number of clones were characterized in preliminary studies. These identified examples in which the levels of constitutively expressed HA-CLIP-hM_3_RASSL remained constant while expression of varying levels of VSV-SNAP-hM_2_WT could be achieved by cell maintenance in the presence of different concentrations of doxycycline. A representative clone is shown in Fig. 5. Cell surface VSV-SNAP-hM_2_WT and HA-CLIP-hM_3_RASSL were imaged individually following addition of the cell impermeant dyes SNAP-surface 549 or CLIP-surface 488. As shown in Fig. 5*A* the CLIP-tagged receptor was present both with and without doxycycline treatment while the SNAP-tagged receptor was only present following doxycycline treatment. Merging of these images indicated clear co-localization of the two receptors at the resolution of light microscopy (Fig. 5*A*). Levels of binding of CLIP-Lumi4Tb (reflecting the presence of HA-CLIP-hM_3_RASSL) to these cells were constant over a range of doxycycline concentrations. By contrast, binding of SNAP-Lumi4Tb (reflecting the appearance of VSV-SNAP-hM_2_WT) increased with increasing concentrations of doxycycline (Fig. 5*B*). To better quantify the relative levels of VSV-SNAP-hM_2_WT and HA-CLIP-hM_3_RASSL expression we measured the specific binding of [^3^H]QNB. Concentrations (16–21.6 nm in individual experiments) were calculated to occupy some 87–90% of HA-CLIP-hM_3_RASSL and more than 98% of VSV-SNAP-hM_2_WT in membranes prepared from cells treated or not with doxycycline. This defined that HA-CLIP-hM_3_RASSL was present at 1632 ± 650 fmol·mg protein^−1^. Moreover, because after treatment with 5 ng·ml^−1^ doxycycline the combined level of expression of muscarinic receptors was 4678 ± 1481 fmol·mg protein^−1^ (Fig. 5*C*), these studies indicated the hM_2_WT could be expressed at up to twice the total level of hM_3_RASSL. In parallel sets of immunoblots of SDS-PAGE-resolved samples, anti-VSV antibodies only detected protein of the appropriate molecular mass, corresponding to VSV-SNAP-hM_2_WT, following treatment of the cells with doxycycline (Fig. 5*D*). Immunoblots using the combined anti-SNAP/CLIP antiserum confirmed that a polypeptide(s) in the region of 80 kDa (VSV-SNAP-hM_2_WT) was expressed in a doxycycline-dependent manner by these cells, while a polypeptide(s) in the region of 110 kDa (HA-CLIP-hM_3_RASSL) was expressed constitutively (Fig. 5*D*).

**FIGURE 6.**
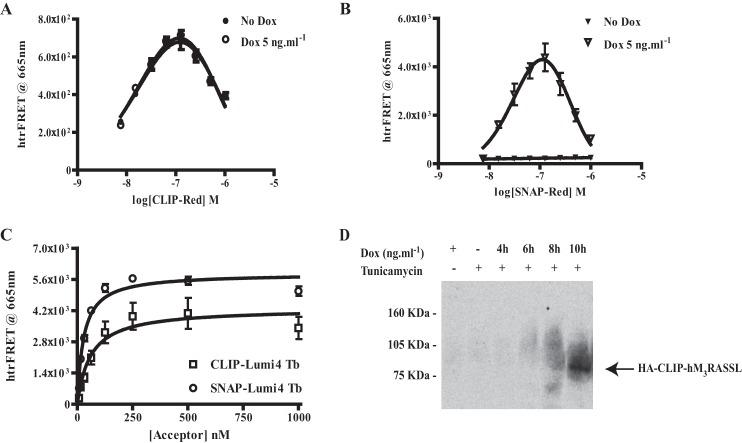
**Detection of homomers of both hM_2_WT and hM_3_RASSL as well as hM_2_WT-hM_3_RASSL heteromers in cells expressing both muscarinic receptor subtypes.** Cells, as in Fig. 5, expressing HA-CLIP-hM_3_RASSL in a constitutive manner and able to express VSV-SNAP-hM_2_WT in a doxycycline-dependent fashion were employed. *A*, htrFRET studies using combinations of CLIP-Lumi4Tb and CLIP-Red demonstrate the presence of hM_3_RASSL homomers in both the absence (*filled symbols*) and presence (*open symbols*) of hM_2_WT. *B*, htrFRET studies using combinations of SNAP-Lumi4Tb and SNAP-Red demonstrate the presence of hM_2_WT homomers only after treatment with doxycycline (*open symbols*) and the expression of VSV-SNAP-hM_2_WT receptor. No such interactions were detected without receptor induction (*filled symbols*). *C*, addition of combinations of either SNAP-Lumi4Tb and CLIP-Red (*circles*) or CLIP-Lumi4Tb and SNAP-Red (*squares*) followed by htrFRET analysis shows also the presence of hM_2_WT-hM_3_RASSL heteromers when the two receptor subtypes are co-expressed. *D*, lysates of untreated cells (−*Dox*) or those treated with doxycycline (+) for various periods and maintained in the presence of the *N*-glycosylation inhibitor tunicamycin (*Tun*) were immunoprecipitated with anti-VSV. These samples were then resolved by SDS-PAGE and immunoblotted with anti-HA to detect co-immunoprecipitation of HA-CLIP-hM_3_RASSL. As induction of expression of VSV-SNAP-hM_2_WT requires a significant period of time after addition of doxycycline, co-immunoprecipitation is only observed at the later time points. *Note:* As shown in Fig. 1, anti-HA is only able to identify the non-N-glycosylated form of HA-CLIP-hM_3_RASSL. This is why experiments were performed in tunicamycin-treated cells.

Using these cells, without doxycycline treatment, homomeric HA-CLIP-hM_3_RASSL interactions were clearly detected as htrFRET signal at 665 nm following addition of combinations of CLIP-Lumi4Tb and CLIP-Red (Fig. 6*A*). Interestingly, such interactions were maintained when the VSV-SNAP-hM_2_WT construct was also expressed, *i.e.* following treatment with doxycycline (Fig. 6*A*). By contrast, and as anticipated, no htrFRET signal corresponding to VSV-SNAP-hM_2_WT homomers was detected in the absence of doxycycline, because this receptor is absent. However, htrFRET signal corresponding to VSV-SNAP-hM_2_WT homomers appeared at the cell surface following doxycycline treatment of the cells (Fig. 6*B*). Importantly, in the doxycycline-induced cells addition of combinations of SNAP-Lumi4Tb and CLIP-Red also demonstrated the proximity of hM_2_WT and hM_3_RASSL, potentially within heteromeric oligomers (Fig. 6*C*). Moreover, hM_2_WT-hM_3_RASSL hetero-interactions were also detected when the labeling protocol was reversed to use a combination of CLIP-Lumi4Tb and, therefore, HA-CLIP-hM_3_RASSL as the energy donor, and SNAP-Red and, therefore, VSV-G-SNAP hM_2_WT as energy acceptor (Fig. 6*C*). Immunoprecipitation of VSV-SNAP-hM_2_WT with anti-VSV antibodies resulted in co-immunoprecipitation of anti-HA immunoreactivity, corresponding to HA-CLIP-hM_3_RASSL, only after doxycycline treatment had resulted in the co-expression of the two receptors (Fig. 6*D*).

**FIGURE 7.**
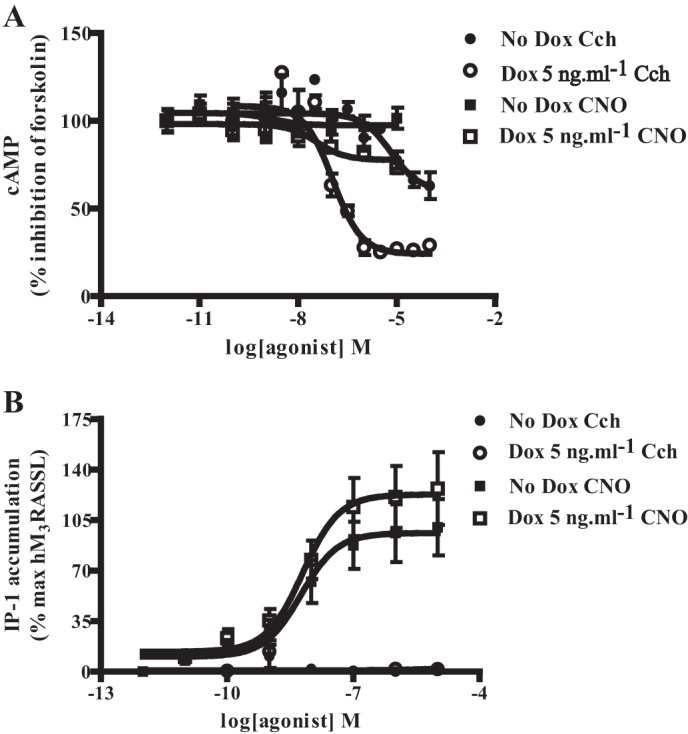
**hM_2_WT and hM_3_RASSL constructs display the anticipated pharmacological selectivity.** Cells constitutively expressing HA-CLIP-hM_3_RASSL and harboring VSV-SNAP-hM_2_WT at the doxycycline-inducible locus were maintained in the absence (*filled symbols*) or presence (*open symbols*) of 5 ng·ml^−1^ doxycycline for 24 h. Subsequently cells were employed to measure the ability of carbachol (*Cch*) (*circles*) or CNO (*squares*) to produced inhibition of forskolin-stimulated cAMP levels (*A*) or the capacity of either CNO or carbachol to mediate increases in levels of inositol monophosphates (*B*).

hM_2_ is linked predominantly to Pertussis toxin-sensitive, G_i_-family G proteins while hM_3_ is usually largely associated with signaling via G_q/11_-family G proteins. Moreover, although the acetylcholine mimetic carbachol is able to activate WT muscarinic receptors, it is reported to display very low potency at RASSL forms of this receptor family (23, 24). This was confirmed in cells induced to express VSV-SNAP hM_2_WT in the constitutive presence of HA-CLIP-hM_3_RASSL. Here carbachol was able to effectively inhibit forskolin-stimulated cAMP production with pEC_50_ = 6.9 ± 0.1 (mean ± S.E., *n* = 4). However, in cells not induced to express VSV-SNAP-hM_2_WT and, therefore, with only HA-CLIP-hM_3_RASSL present, little inhibition of forskolin-stimulated cAMP levels was noted at concentrations of carbachol up to 1 μm (Fig. 7*A*). By contrast, both in the absence (pEC_50_ = 8.10 ± 0.08) and presence (pEC_50_ = 8.00 ± 0.18) (means ± S.E., *n* = 5 in each case) of VSV-SNAP hM_2_WT, CNO was able to potently stimulate the production of inositol monophosphates (Fig. 7*B*). This is a downstream indicator of G_q_/G_11_ activation. Interestingly, although not reaching statistical significance, there was a trend toward higher inositol monophosphate production in response to CNO when the two receptors were co-expressed (Fig. 7*B*). This did not reflect a direct effect of CNO on the hM_2_WT receptor orthosteric binding pocket because neither with nor without doxycycline induction was carbachol able to cause a significant accumulation of inositol monophosphates in these cells (Fig. 7*B*). Importantly, however, these studies did define the functionality of the expressed constructs and confirmed the previously established selectivity of the agonist ligands in this setting (23, 24).

**FIGURE 8.**
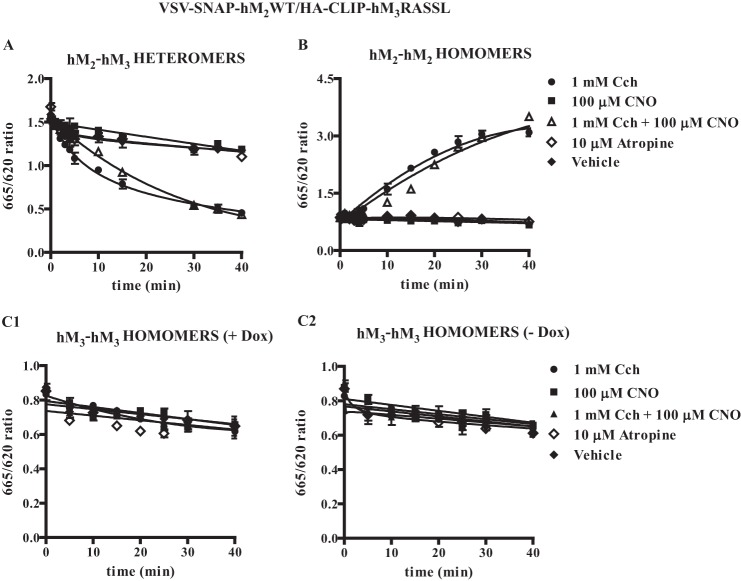
**Carbachol but not Clozapine N-Oxide disrupts hM_2_WT-hM_3_RASSL heteromers and enhances hM_2_WT homomer interactions.** *A* and *B*, htrFRET studies were performed as in Fig. 6 in cells induced to co-express VSV-SNAP-hM_2_WT and HA-CLIP-hM_3_RASSL and detected signals consistent with each of hM_2_WT-hM_3_RASSL heteromers (*A*) or hM_2_WT homomers (*B*). Ligands selective for the hM_2_WT (*carbachol, Cch*) or hM_3_RASSL (*CNO*) were added for the noted times and htrFRET signal recorded. In other experiments the non-subtype selective muscarinic antagonist *atropine* or *vehicle* was added. Carbachol selectively diminished htrFRET signal corresponding to hM_2_WT-hM_3_RASSL heteromer interactions (*A*) while increasing signal corresponding to hM_2_WT homomeric interactions (*B*). *C*, equivalent studies were performed on cells expressing either both hM_2_WT and hM_3_RASSL (*C1*) or hM_3_RASSL alone (*C2*) and were designed to detect the hM_3_RASSL homomer. No ligand-specific effects were noted on the quaternary organization of this receptor complex.

**FIGURE 9.**
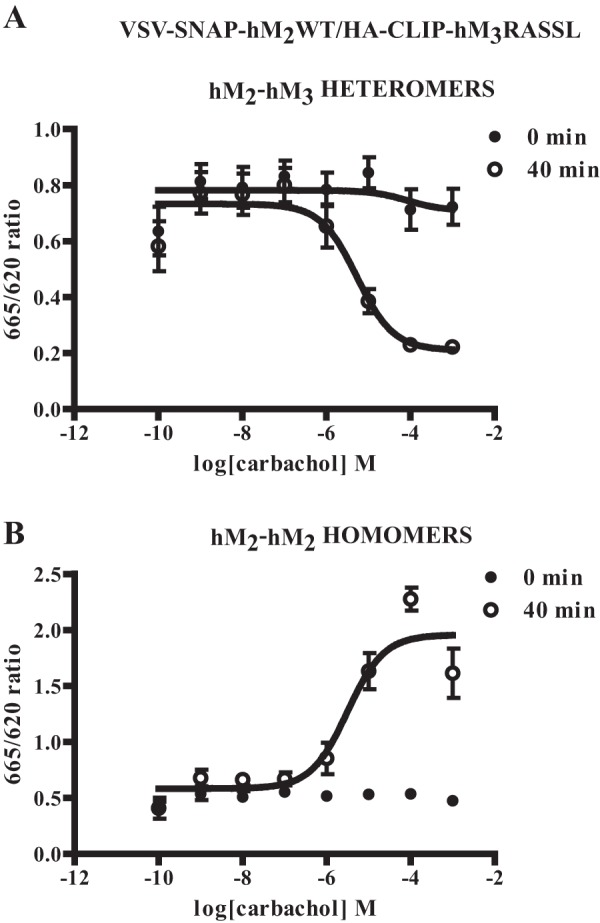
**Effects of carbachol on muscarinic receptor quaternary organization are concentration-dependent.** Experiments akin to those of Fig. 8 were performed using cells induced to co-express VSV-SNAP-hM_2_WT and HA-CLIP-hM_3_RASSL and detected signals consistent with each of hM_2_WT-hM_3_RASSL heteromers (*A*) or hM_2_WT homomers (*B*). The effect of varying concentrations of carbachol was assessed immediately after addition or 40 min later. Data represent means ± S.E., *n* = 4.

Potential effects of ligands on the organization or stability and regulation of GPCR oligomers is a complex topic in which a range of observations have been reported (11). In cells induced with doxycycline to allow co-expression of VSV-SNAP-hM_2_WT and HA-CLIP-hM_3_RASSL, as noted above, co-addition of a combination of SNAP-Lumi4Tb and CLIP-Red resulted in detection of htrFRET signal, consistent with interactions between the two receptors (Fig. 8*A*). Over a period of 40 min, exposure to a concentration (10 μm) of the muscarinic antagonist atropine that is sufficient to occupy fully both the hM_2_WT and hM_3_RASSL constructs, had no greater effect on the heteromer signal than addition of vehicle (Fig. 8*A*). By contrast, addition of either carbachol alone, or a combination of carbachol and CNO, resulted in a substantial and rapid decline in htrFRET signal corresponding to hM_2_WT-hM_3_RASSL interactions (Fig. 8*A*). Unlike carbachol, CNO was unable to produce such an effect when applied alone (Fig. 8*A*). When equivalent studies were performed using a combination of SNAP-Lumi4Tb and SNAP-Red to detect hM_2_ receptor homomers, both carbachol alone and carbachol plus CNO now resulted instead in an extensive increase in htrFRET signal (Fig. 8*B*). Once again neither CNO alone, nor atropine had any effect on the hM_2_ homomer htrFRET signal compared with vehicle-treated cells (Fig. 8*B*). Unlike the hM_2_WT homomer, htrFRET signal corresponding to the hM_3_RASSL homomer was not affected in these cells in a ligand-dependent manner (Fig. 8*C*). This was the case whether or not expression of VSV-SNAP-hM_2_WT had been induced (Fig. 8*C*). Importantly, the effects of carbachol on both the hM_2_WT homomer (pEC_50_ = 5.5 ± 0.2) and hM_2_WT-hM_3_RASSL heteromer (pEC_50_ = 5.2 ± 0.3) (means ± S.E., *n* = 4 in each case) interactions were concentration-dependent (Fig. 9).

**FIGURE 10.**
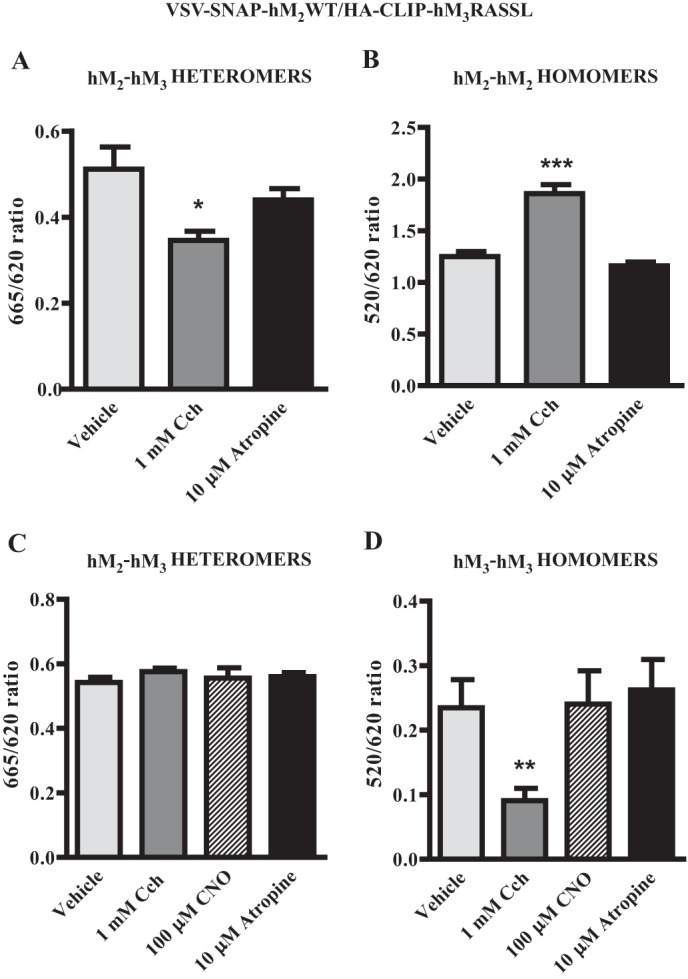
**Concurrent detection of the presence hM_2_WT homomers and hM_2_WT-hM_3_RASSL heteromers and their regulation by carbachol.** Cells as in Fig. 9 induced to co-express VSV-SNAP-hM_2_WT and HA-CLIP-hM_3_RASSL were incubated with a combination of each of SNAP-Lumi4Tb, CLIP-Red, and SNAP-Green (*A, B*) and htrFRET signal measured at both 665 nm (SNAP-Lumi4Tb to CLIP-Red (hM_2_WT-hM_3_RASSL heteromer)) (*A*) and 520 nm (SNAP-Lumi4Tb to SNAP-Green (hM_2_WT homomer)) (*B)*. Treatment with carbachol but not atropine reduced the hM_2_WT-hM_3_RASSL heteromer signal (*A*) and concurrently increased the hM_2_WT homomer signal (*B*). Equivalent studies used a combination of CLIP-Lumi4Tb and both SNAP-Green and CLIP-Red (*C, D*). Treatment with carbachol only reduced the hM_2_WT-hM_3_RASSL heteromer (520 nm) signal (*C*) while neither carbachol, atropine nor CNO had any effect on the hM_3_RASSL homomer (665 nm) signal (*D*). Data represent means ± S.E., *n* =3. Statistical significance as follows: *, *p* < 0.05, **, *p* < 0.001, and ***, *p* < 0.0001 when compared with vehicle.

As an extension to these studies we attempted to identify concurrently in the same cells both homo- and hetero-interactions involving VSV-SNAP-hM_2_WT. SNAP- and CLIP-Lumi4Tb have broad emission spectra. As such, upon excitation at 337 nm they can potentially transfer energy to both Green (with htrFRET output at 520 nm) and Red (with htrFRET output at 665 nm) energy acceptors. This potentially allows concurrent dual color detection of multiple interactions of the energy donor-tagged receptor. We, therefore, initially added a mixture of SNAP-Lumi4Tb and both CLIP-Red and SNAP-Green to cells induced to co-express VSV-SNAP-hM_2_WT and HA-CLIP-hM_3_RASSL. Such studies were indeed able to identify interactions of the energy donor-labeled hM_2_WT with both energy acceptor labeled hM_2_WT and hM_3_RASSL receptors concurrently (Fig. 10, *A* and *B*). Moreover, as in the individual htrFRET experiments reported above, concurrent analysis of the two distinct interactions of the energy donor-labeled hM_2_WT receptor showed an equivalent carbachol-mediated decrease in hM_2_WT-hM_3_RASSL heteromeric interactions (Fig. 10*A*) and increase in hM_2_WT-hM_2_WT homomeric interactions (Fig. 10*B*). Once again, the muscarinic antagonist atropine was without effect (Fig. 10, *A* and *B*). Finally, in cells induced to co-express the hM_2_WT and hM_3_RASSL receptors, labeling of hM_3_RASSL with the energy donor CLIP-Lumi4Tb and proportions of both hM_2_WT and hM_3_RASSL respectively with SNAP-Green and CLIP-Red, the hM_2_WT-hM_3_RASSL heteromeric interactions were again specifically decreased by treatment with carbachol (Fig. 10*C*). By contrast hM_3_RASSL homo-interactions were once more unperturbed by addition of any of CNO, carbachol, or atropine (Fig. 10*D*).

## Discussion

Although it is well established that monomers of the individual subtypes of muscarinic acetylcholine receptors can exist in proximity to one another (4, 26–28) and, indeed, have the capacity to generate dimers and/or higher-order oligomers (6–7, 9, 18, 27–28), a broad range of issues around such interactions remain unresolved. Among these are the stability (19) or otherwise (8, 18) of dimeric interactions, the overall dimensions and organization of dimeric/oligomeric complexes (7, 9, 25) and the implications of this for the details of interaction with heterotrimeric G proteins and downstream signal transduction (11–12). Moreover, as muscarinic subtypes are expressed at markedly different levels in different cells and tissues this may, as suggested by some (25) but not other (19–20) reports on both muscarinic and other rhodopsin-like family GPCRs, affect the extent of their dimerization/oligomerization. Furthermore, distinct muscarinic receptor subtypes may be co-expressed in physiologically relevant cells (29–30). Although the capacity for heteromeric interactions between various muscarinic receptor pairs has been explored to some degree (26–27), the propensity for this to occur concurrently with homomerization, and its implications for function, have been little explored to date. For example, M_2_ and M_3_ receptors are co-expressed in smooth muscle but the functional importance of this for the integration of signaling remains uncertain. Within the current studies we have, therefore, addressed a number of these issues by combinations of biochemical, biophysical, and chemical biology approaches.

Central to these studies was the use of cell surface hrtFRET, based on the incorporation of SNAP- and CLIP-tags (31–32), into various muscarinic receptor constructs. Such tagging allowed the covalent incorporation of hrtFRET-competent fluorophores into the extracellular N-terminal region of the receptors via linkage to the engineered SNAP and CLIP protein tags. Importantly, such large scale modification of the N-terminal domain of either the hM_2_ or hM_3_ receptor did not affect their basic ligand pharmacology. Of equal importance was the introduction of RASSL-inducing mutations into the hM_3_ receptor constructs (23–24). Particularly for the muscarinic receptor family, such modified GPCRs are also frequently denoted as DREADDs (Designer Receptors Exclusively Activated by Designer Drugs) (33). The associated alteration in agonist pharmacology so produced allowed for selective agonist occupancy and activation of the hM_2_WT receptor (with the acetylcholine mimetic carbachol) and the hM_3_RASSL receptor (with the muscarinic RASSL agonist CNO) in cells co-expressing the two receptor subtypes. This was confirmed by demonstrating both that carbachol-mediated inhibition of cAMP levels was observed only following induced expression of the hM_2_ receptor and not when the hM_3_RASSL receptor was expressed alone, and that CNO, but not carbachol, was able to promote the production of inositol monophosphates via the hM_3_RASSL receptor, both in the absence and presence of the hM_2_ WT receptor. By contrast, the antagonist atropine was able to bind to both receptors with similar affinity.

In cells able to express only either the SNAP-tagged hM_2_WT receptor or the CLIP-tagged RASSL form of the hM_3_ receptor htrFRET studies provided clear evidence for homomeric interactions of each subtype. Although this was anticipated from previous work, in cells constitutively expressing hM_3_RASSL receptors, induced expression of the hM_2_WT receptor now resulted in detection of hM_2_-hM_2_ interactions as well as hM_2_-hM_3_ interactions at the surface of these cells without eliminating hM_3_-hM_3_ interactions. The most obvious interpretation of these results is that receptor homomers can co-exist with relevant heteromers perhaps, as suggested by Herrick-Davis *et al.*, as stable and distinct dimers (20). However, it is important to note that others have suggested such interactions to be more dynamic (18, 34). Moreover, concurrent monitoring of hM_2_-hM_2_ and hM_2_-hM_3_ interactions in dual color studies, in which a single energy donor and two distinct energy acceptor reagents were added concurrently, also provided evidence for each of these interactions. This is the first time that such an approach has been used to examine multiple interaction partners of a GPCR simultaneously.

A common concern in studies on interactions involving cell surface transmembrane proteins is that high level expression may result in apparent interactions based on proximity that reflect the levels of expression achieved. We addressed this in two distinct ways. Firstly, for all the studies performed we generated and utilized stably transfected cell lines able to express the receptor(s) of interest in a controlled, inducible manner. Generally, studies that rely entirely on transient transfection protocols encounter challenges due to high level expression of the receptors, often incompletely processed, within subsetsof the cell population. Herein, we demonstrated that the bulk of each of the muscarinic receptor subtype constructs was appropriately *N*-glycosylated, as anticipated for mature, correctly trafficked GPCRs. More importantly we also generated an equivalent cell line able to inducibly express VSV-SNAP-CD86. CD86 is recognized as a monomeric single transmembrane domain protein (25). Expression of this construct to the same level as used to study VSV-SNAP-hM_2_WT generated no specific htrFRET signal upon addition of a combination of SNAP-tag energy donor and acceptor species. This provided comfort that the signals produced at these levels of expression of VSV-SNAP-hM_2_WT did indeed reflect true receptor-receptor interactions.

The further key outcome of these studies is that the agonist carbachol was able to change energy transfer signals corresponding to both hM_2_-hM_2_ and hM_2_-hM_3_ interactions. By contrast this ligand had no effects on hM_3_-hM_3_ interactions. This latter feature was hardly surprising as the hM_3_RASSL constructs used in these studies were modified to have minimal affinity for carbachol (23–24). However, in the case of the hM_2_-hM_2_ and hM_2_-hM_3_ interactions the directionality of the effect of carbachol was completely different. Both in cells expressing only the hM_2_WT receptor construct, and those expressing both the hM_2_WT and the hM_3_RASSL receptors, carbachol increased the htrFRET signal corresponding to hM_2_-hM_2_ homomers and did so in both a time- and concentration-dependent manner. Moreover, the EC_50_ for the ligand in producing these changes in htrFRET was very similar to the affinity of carbachol at the hM_2_WT receptor. This is consistent with the effects reflecting receptor occupancy. By contrast carbachol decreased the htrFRET signal corresponding to hM_2_WT-hM_3_RASSL interactions. This was, however, once again both time- and concentration-dependent. It could be argued in the hM_2_-hM_3_ co-expressing cells that the effect of carbachol was to diminish hM_2_-hM_3_ interactions and that this then resulted in greater hM_2_-hM_2_ interactions, *i.e.* to promote a heteromer to homomer transition. However, although these effects of carbachol could also be detected in triple labeling, 'dual color' studies in which the effects on the receptor complexes were measured concurrently, further studies will be required to support such a conclusion. Perhaps surprisingly, unlike carbachol, CNO was unable to influence htrFRET signals corresponding to hM_3_RASSL-hM_3_RASSL interactions to any greater extent than addition of vehicle. This may reflect greater stability of hM_3_-hM_3_ homomeric interactions compared with either hM_2_-hM_2_ homomers or hM_2_-hM_3_ heteromers. However, although identified as a highly selective activator of RASSL forms of muscarinic receptor subtypes, CNO is of course not a direct equivalent of carbachol. This is despite CNO acting as an apparently high efficacy agonist that, in a wide range of assays, shows broad similarity in capacity to activate and regulate the hM_3_RASSL as either carbachol or acetylcholine do at the wild type hM_3_ receptor (23). Although there may be differences in details of efficacy or bias of CNO at the hM_3_RASSL receptor in end points that have not been assessed previously that may account for this difference, a distinct explanation is that the hM_2_ and hM_3_ receptors differ in the basis or stability of their homomeric interactions. It is notable in this regard that Calebiro *et al.* have provided evidence for markedly different stability and propensity of β_1_- and β_2_-adrenoceptors to form dimers and higher-order oligomers (25), even though these receptors are highly homologous and are activated by the same hormones.

This is not the first set of studies to suggest a capacity of ligand to alter the organization and/or stability of a muscarinic receptor homomer. Although muscarinic toxin 7, a highly selective allosteric peptide ligand of the M_1_ subtype, binds (35–36) in a very different manner to carbachol or CNO (23), it has been reported to stabilize M_1_ receptor homomers (35–36). It has also been suggested that the selective M_1_ receptor antagonist pirenzepine can promote dimerization of this receptor (37).

Beyond possible differences in efficacy, one further observation that is difficult to provide a clear explanation for was the marked difference in the effects of carbachol and CNO on hM_2_WT-hM_3_RASSL interactions and, thus, on hM_2_-hM_3_ heteromers. Although difficult to demonstrate without making further alterations in the ligand binding pocket to alter ligand pharmacology, as has been done for the β_2_-adrenoreceptor (38) and the leukotriene B(4) receptor (39), which, to some extent invalidates the basis of the experiment, it is anticipated that a ligand effect across the interface of a receptor homo-dimer/oligomer should be symmetric. Therefore, an effect of ligand binding to one protomer is anticipated to be reciprocated by (the same) agonist occupancy of the other protomer. Herein, carbachol effects on hM_2_WT-hM_3_RASSL receptor interactions were not recapitulated by CNO. This may simply reflect that the hM_2_ and hM_3_ receptors are, of course, distinct species or that the makeup of hM_2_WT-hM_3_RASSL receptor heteromers is not simply 1:1 in oligomeric (7, 28) rather than dimeric configurations. No-matter the basis for the lack of symmetry here, this is topic that requires and deserves further consideration in the future.

Notwithstanding this final point, the current studies offer a broad range of novel insights into differences in ligand regulation of hM_2_-hM_2_ *versus* hM_3_-hM_3_ interactions and provide a one donor plus two acceptors strategy to concurrently assess interactions of a protein with more than a single partner. The molecular basis for the noted differences in ligand regulation between closely related receptors will provide a drive for future analysis.

## Footnotes

3 The abbreviations used are:

GPCR: G protein-coupled receptor
CNO: clozapine N-oxide
DDM: n-dodecyl-β-d-maltoside
DREADD: Designer Receptors Exclusively Activated by Designer Drug
htrFRET: homogeneous time-resolved Fluorescence Resonance Energy Transfer
QNB: quinuclidinylbenzilate
RASSL: Receptor Activated Solely by Synthetic Ligand.
